# Subthalamic nucleus activity dynamics and limb movement prediction in Parkinson’s disease

**DOI:** 10.1093/brain/awz417

**Published:** 2020-02-10

**Authors:** Saed Khawaldeh, Gerd Tinkhauser, Syed Ahmar Shah, Katrin Peterman, Ines Debove, T A Khoa Nguyen, Andreas Nowacki, M Lenard Lachenmayer, Michael Schuepbach, Claudio Pollo, Paul Krack, Mark Woolrich, Peter Brown

**Affiliations:** 1 MRC Brain Network Dynamics Unit, University of Oxford, UK; 2 Nuffield Department of Clinical Neurosciences, University of Oxford, UK; 3 Oxford Centre for Human Brain Activity, Wellcome Centre for Integrative Neuroimaging, University of Oxford, UK; 4 Department of Neurology, Bern University Hospital and University of Bern, Switzerland; 5 Usher Institute of Population Health Sciences and Informatics, Edinburgh Medical School, The University of Edinburgh, Edinburgh, UK; 6 Department of Neurosurgery, Bern University Hospital and University of Bern, Switzerland

**Keywords:** deep brain recording, brain computer interface, subthalamic nucleus, machine learning, Parkinson’s disease

## Abstract

Whilst exaggerated bursts of beta frequency band oscillatory synchronization in the subthalamic nucleus have been associated with motor impairment in Parkinson’s disease, a plausible mechanism linking the two phenomena has been lacking. Here we test the hypothesis that increased synchronization denoted by beta bursting might compromise information coding capacity in basal ganglia networks. To this end we recorded local field potential activity in the subthalamic nucleus of 18 patients with Parkinson’s disease as they executed cued upper and lower limb movements. We used the accuracy of local field potential-based classification of the limb to be moved on each trial as an index of the information held by the system with respect to intended action. Machine learning using the naïve Bayes conditional probability model was used for classification. Local field potential dynamics allowed accurate prediction of intended movements well ahead of their execution, with an area under the receiver operator characteristic curve of 0.80 ± 0.04 before imperative cues when the demanded action was known ahead of time. The presence of bursts of local field potential activity in the alpha, and even more so, in the beta frequency band significantly compromised the prediction of the limb to be moved. We conclude that low frequency bursts, particularly those in the beta band, restrict the capacity of the basal ganglia system to encode physiologically relevant information about intended actions. The current findings are also important as they suggest that local subthalamic activity may potentially be decoded to enable effector selection, in addition to force control in restorative brain-machine interface applications.

## Teaser

Parkinson’s disease bursts of oscillatory synchronization in the beta band are associated with motor impairment. Khawaldeh *et al.* provide clues to the underlying mechanisms by showing that beta bursts restrict the capacity of the basal ganglia system to encode physiologically relevant information about intended actions.

## Introduction

Beta activity is a prominent feature recorded in the basal ganglia of patients with Parkinson’s disease and in animal models of the condition (Brown *et al.*, 2001; Mallet *et al.*, 2008; Deffains *et al.*, 2016). It reflects excessive synchronization within basal ganglia circuits (Levy *et al.*, 2002*a*, *b*; Kühn *et al.*, 2005; Weinberger *et al.*, 2006; Cagnan *et al.*, 2019) and its central role in parkinsonism is recognized in many computational models of the disease (van Albada *et al.*, 2009; Moran *et al.*, 2011; Marreiros *et al.*, 2013; Pavlides *et al.*, 2015; Müller and Robinson, 2018). Beta activity is attenuated by the dopamine prodrug, levodopa, and by deep brain stimulation (DBS) of the subthalamic nucleus (STN), a key surgical target in the basal ganglia (Brown *et al.*, 2001; Priori *et al.*, 2004; Kühn *et al.*, 2008). The degree of beta activity correlates with motoric impairment, and the degree of suppression of beta activity by drugs or DBS correlates with the level of improvement in motoric impairment (Kühn *et al.*, 2006, 2008; Ray *et al.*, 2008; Neumann *et al.*, 2016; Oswal *et al.*, 2016; Steiner *et al.*, 2017). Recently, it has become evident that beta activity in Parkinson’s disease comes in bursts of over 100 ms in duration (Tinkhauser *et al.*, 2017*a*, *b*; Deffains *et al.*, 2018) and that the aforesaid clinical correlations may be even stronger when considering the propensity for such bursts (Torrecillos *et al.*, 2018; Lofredi *et al.*, 2019). That beta bursts may be causally important is suggested by the therapeutic efficacy of using high frequency stimulation to selectively target such bursts (Little *et al.*, 2013; Tinkhauser *et al.*, 2017*a*).

However, the link between synchronization in the beta band and motoric impairment is somewhat paradoxical given current and well-established theories that ascribe improved communication and more efficient neural processing to oscillatory synchrony (Engel *et al.*, 2001; Salinas and Sejnowski, 2001; Varela *et al.*, 2001; Buzsáki and Draguhn, 2004; Fries, 2005, 2015). One solution to this paradox may be that the performance of a neuronal circuit may deteriorate with too much synchronization, as the restriction in information coding capacity outstrips the improvements in signal-to-noise ratio engendered by synchronization (Brittain and Brown, 2014). Support for this hypothesis comes from studies showing an increase in mutual information between different channels in the STN in animal models of parkinsonism compared to the healthy state (Mallet *et al.*, 2008). However, mutual information is an information theoretic measure that only helps dictate the upper bound of the possible information that can be held by a system; the greater the mutual information across its components the less the information coding capacity of the system. However, mutual information does not necessarily capture those aspects of circuit activity that reflect genuine neural processing as suggested by their association with forthcoming actions.

Here we test the hypothesis that periods of elevated oscillatory synchrony, as marked by bursts in the local field potential (LFP) activity of the STN, impair the local representation of task-related information in the STN. To this end we use directional electrodes allowing a high resolution read-out of changing features in the STN LFP (Zhang *et al.*, 2018), and machine learning to identify the aspects of wide-band STN LFP activity that predict whether a voluntary upper or lower limb movement will subsequently be performed, either because the identified features are directly important in neural processing or because they are informative surrogates of such processing. Our findings help provide a mechanistic explanation for the correlative findings linking bursts of low frequency activity to parkinsonian motor impairment, while also focusing attention on the STN as a potential alternative or complementary signal source for restorative brain-machine interfaces.

## Materials and methods

### Patients and surgery

**Figure 1:**
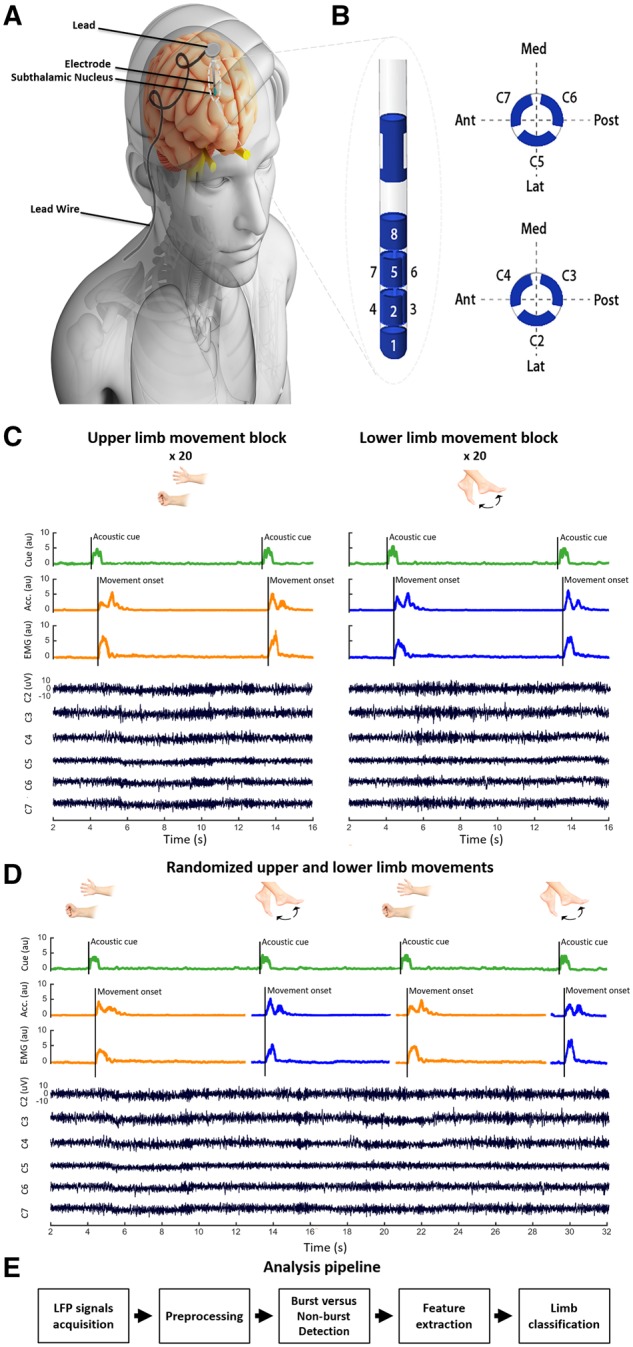
**Experimental setup and analysis pipeline.** (**A**) Deep brain electrode schematic. (**B**) The directional DBS lead (Boston Scientific). Contacts are distributed along four levels. On levels two and three, there are three segmented contacts (level two: contacts 2/3/4; level three: contacts 5/6/7). (**C**) The fixed-limb voluntary movement task; upper and lower limb movements performed in separate blocks, with each block preceded with an instruction describing the limb to be moved after hearing the imperative auditory cue. (**D**) Random-limb voluntary movement task; upper and lower limb movements randomly instructed within the same experimental block. Here the auditory cue prior to each trial also describes the limb to be moved. (**E**) Flow chart summarizing analysis pipeline. LFP signals are used to predict the limb moved using the naïve Bayes technique. Acc. = acceleration; Ant = anterior; au = arbitrary units; Lat = lateral; Med = medial; Post = posterior.

We studied the task-related modulation of STN LFPs before and during voluntary upper and lower limb movements in 18 consecutive Parkinson’s disease patients undergoing STN DBS surgery to improve motor symptoms (clinical details are provided in Supplementary Table 1). Recordings were made intraoperatively from both hemispheres, except in seven subjects (Cases 5, 6, 9, 11, 15, 16 and 18) in whom LFPs were recorded in one hemisphere only, due to fatigue and operative constraints. Thus, a total of 29 hemispheres were studied. Patients were recorded OFF dopaminergic medication. All patients were operated at the University Hospital Bern and the local ethics committee approved the use of the data for scientific purposes (2017-00551). Patients were implanted with Boston Vercise Cartesia directional electrodes (Boston Scientific). The contacts of these electrodes are distributed along four vertical levels as shown in Fig. 1B. The middle two levels each contain three segmented (non-circular) contacts, allowing stimulation and recording focussed in three different directions (each at 120° angles). The top and bottom levels consist of a single ring/omnidirectional contact each. Localization of the STN was performed using the T_2_-sequence of the preoperative 3 T MRI and preoperative stereotactic CT scan (with Leksell G frame) assisted by Brainlab iPlan 3.0 Stereotaxy software (Brainlab AG, Germany). Intraoperative targeting was optimized by microelectrode recordings and selective test stimulation.

### Postoperative localization of directional contacts

**Figure 2:**
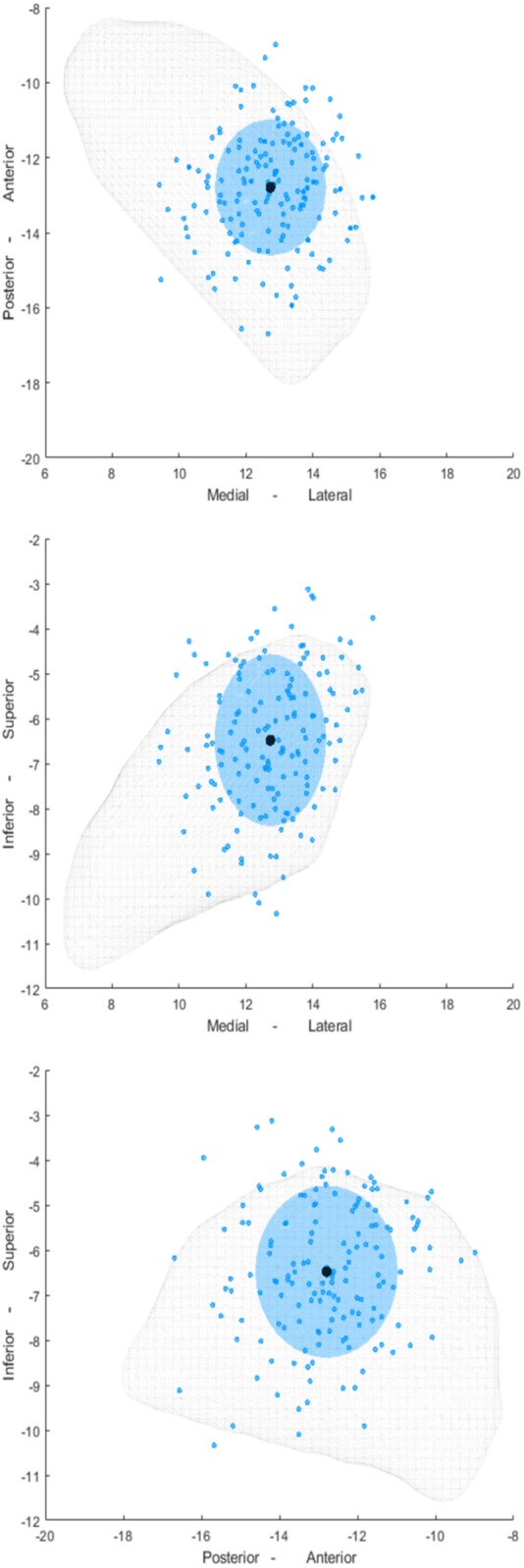
**Localization of all the directional contacts in all 18 subjects included in the study.** Distribution of the contacts (blue dots) and the mean coordinate of these contacts (black dots) are shown relative to the STN (grey mesh) in three different planes. In addition, a blue-shaded sphere is shown where the diameter is separately defined for the *x, y,* and *z* coordinates and corresponds to a range of 2.5 standard deviations.

To visualize the distribution of the directional contacts in the STN we used the Lead-DBS MATLAB toolbox (version 2.1.6) (Horn *et al.*, 2019). Preoperative MRI and postoperative CT scans were co-registered using Advanced Normalization Tools (Avants *et al.*, 2008) and SPM12 (Statistical Parametric Mapping 12; Wellcome Trust Centre for Neuroimaging, UCL, London, UK) and normalized into the MNI 152 2009b space (Montreal Neurological Institute) (Avants *et al.*, 2008). Using the Precise and Convenient Electrode Reconstruction for DBS (PaCER) toolbox, the DBS lead was pre-localized and eventually manually adjusted (Husch *et al.*, 2018). Finally, the *x, y, z* coordinates of all directional contacts, from the left and right STN, were projected on to the right STN of the DISTAL Atlas (Ewert *et al.*, 2018) using a non-linear flip function (Lead-DBS MATLAB toolbox) as shown in Fig. 2.

### Local field potential recordings and limb assessments

The general experimental set-up has been previously published (Tinkhauser *et al.*, 2019) and is summarized below and in Fig. 1A–D. After the first electrode was implanted, LFPs were recorded simultaneously from the six directional contacts (contacts 2 to 7, Fig. 1B) at rest and during movement of the contralateral or ipsilateral limbs. Where time allowed, this procedure was then repeated when the second electrode was implanted. A TMSi-Porti amplifier (Twente Medical Systems International) was used for recordings and these were made with a common average reference, low-pass filter of 550 Hz and a sampling frequency of 2048 Hz. Surface EMG electrodes were taped over the forearm flexor muscles of the upper limb and tibialis anterior of the lower limb. Accelerometers were additionally placed on the dorsum of the hand and foot to further enhance detection of movement onset in each task. Finally, the auditory (verbal) cues were captured by a microphone synchronized with the recording system.

After a brief recording at rest (mean duration: 100.8 ± 36.5 s), patients performed one of two tasks. In the first one (fixed-limb blocks, *n* = 12 subjects), patients were instructed to perform separate blocks of upper and lower limb movements. Patients were informed of the desired response at the beginning of each block and instructed that only this movement should be made in response to imperative auditory cues during that block (Fig. 1C). Upper and lower limb blocks were performed in counterbalanced order. In the second task, patients (random-limb blocks, *n* = 4 subjects) were instructed to perform upper and lower limb movements that were randomly intermixed. Here, patients were informed of the desired response at every auditory cue (Fig. 1D), which then acted as a combined instruction and imperative cue. Prior to recording in the two tasks, patients were asked to respond ‘promptly and accurately’.

The upper limb movement consisted of closing and opening of the hand, while the lower limb movement involved ankle dorsi-extension and then plantar flexion (Fig. 1C and D). For the first experiment, each single movement was prompted by a verbal ‘go’ command recorded with the microphone and the intertrial time was 7.7 ± 1.6 s (range: 6.0–11.9 s). This intertrial duration was selected to avoid compromising the baseline period of the next movement with the beta rebound following the last movement (Pfurtscheller and Da Silva, 1999). For the second experiment, each movement was prompted by verbal ‘upper’ and ‘lower’ commands directing movement of the hand or ankle and recorded with the microphone, and the intertrial time was 7 ± 2.1 s (range: 5.5–14.7 s). Our goal was to record 20 trials per STN contralateral to each moved limb per experiment in each subject. However, the exact number of blocks and trials varied due to the intraoperative setting and associated constraints [fixed-limb blocks, 16.54 ± 3.28 trials (range: 10–25 trials); random-limb blocks, 21 ± 11.37 trials (range: 9–40 trials)]. In addition, we were able to record STN activity ipsilateral to upper and lower limb movements in fixed-limb and randomized-limb blocks in 13 and two hemispheres, respectively.

### Signal processing

Spike2 software (CED, Cambridge, UK) was used to manually label the onset of the audio cue, and the onset and end of movements based on the microphone, EMG and accelerometer signals. MATLAB (2018, Mathworks, Natick, MA, USA) was used for segmenting trials, preprocessing and further analyses including training and testing for movement classification. These were performed independently in each subject. OSL [OHBA Software Library; Oxford Centre for Human Brain Activity (OHBA), University of Oxford, Oxford, UK] was used for artefact detection and rejection. EMG signals of upper and lower limbs were extracted, z-scored, rectified, and high-pass filtered at 15 Hz. A general overview of the LFP processing and analysis pipeline is shown in Fig. 1E. The raw signal from each of the six directional electrode contacts was high-pass filtered at 2 Hz, then detrended and *z*-scored to normalize each channel’s data before any trial segmentation or task separation. Frequency decomposition was performed with 1 Hz resolution using the Complex Morlet Wavelet method (Cohen, 2014). A baseline normalization was performed on each segmented trial, where each 1 Hz frequency component in each trial in the time-frequency domain was normalized by a baseline of 500 ms of the same component taken from the beginning of each trial (2 s before auditory cue). Thus, LFPs were expressed as relative change with respect to baseline. This helps normalize changes across hemispheres and subjects, where absolute amplitudes might vary due to targeting variance, differing stun effects and patient’s Parkinson’s disease symptomatology. In two hemispheres (1 and 13), one directional contact had to be excluded from the analysis because of saturation during the recording.

### Feature extraction and selection

A set of 100-ms long non-overlapping windows were used to extract frequency-domain features from the six directional LFP contacts. Normalized LFP powers in nine different frequency bands ranging from 8 to 500 Hz were identified as potential frequency domain features (Shah *et al.*, 2017). These distinct frequency bands were: 8–12 Hz, 13–20 Hz, 21–30 Hz, 31–45 Hz, 56–95 Hz, 106–200 Hz, 201–300 Hz, 301–349 Hz, and 350–500 Hz. After extracting the features, they were standardized to have a zero-mean (by subtracting the mean), and a unit-variance (by dividing by the standard deviation). Low frequency activity (below 7 Hz) was excluded as the 100-ms window used for extracting features was insufficient for reliable estimation. We did, however, explore the additional use of various higher order statistical measures as potential features (entropy, kurtosis, activity, mobility, complexity, skewness, maximum, minimum, mean, and standard deviation; as per Shah *et al.*, 2017) in the classification of the training subsets, but these were seldom prioritized by the feature selection algorithm, and had very low weights compared to the spectral features. As a result, they were excluded from the main analyses.

K-fold cross validation (k: number of groups into which dataset was randomly split) was used to ensure that all trials in the original training dataset were used for both training and validation, and the same subset of data used for training was not used for validation; each trial being used for validation only once. Specifically, we used 4-fold cross-validation, where at each rotation, three folds were used for training, and the remaining one was used for testing. The ReliefF feature selection algorithm was used to determine important features and eliminate non-important ones (Robnik-Šikonja and Kononenko, 2003). ReliefF first sets all feature weights to 0, then iteratively selects a random trial and finds the nearest trials to it from each class, and finally it calculates the weights of features through penalizing weights that give different values to neighbours of the same class, and rewarding those that give different values to neighbours of different classes. This was applied on the training subset containing the three folds, and the top six ranked features used for testing the fourth fold. The weight of each feature was estimated by counting how many times it was selected across the four folds as among the top six features, this was then divided by the number of folds (k = 4) to get the final measure of feature importance. These weights were then used as a measure of the contribution of a given feature to the classification.

### Burst detection

**Figure 3:**
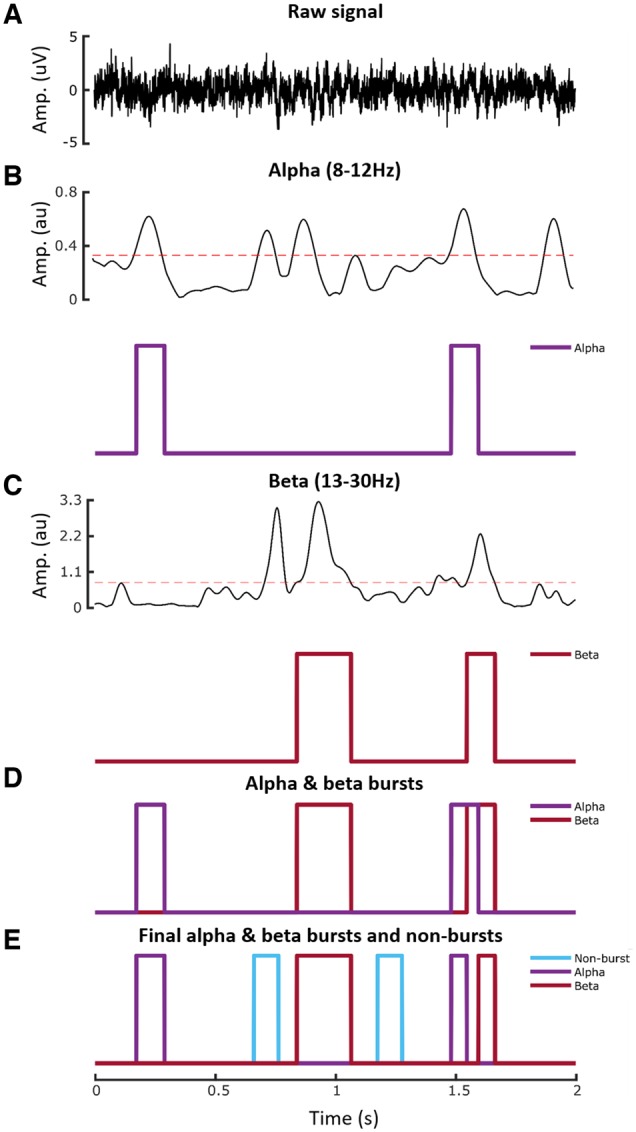
**Burst detection process.** (**A**) Raw LFP in a 2-s epoch from one of the directional contacts. (**B**) LFP power in alpha band and detected alpha bursts. Threshold shown by interrupted red line is the 75th percentile power. Note that threshold crossings had to exceed 100 ms in duration to be classified as bursts. (**C**) LFP power in beta band and detected beta bursts. (**D**) Detected alpha and beta bursts. (**E**) Final non-overlapping alpha and beta bursts, and burst-free periods (non-burst). au = arbitrary units.

Power spectral densities in the alpha (8–12 Hz), beta (13–30 Hz) and low gamma (35–45 Hz) bands were calculated. The 75th percentile thresholding method was used to find bursts which lasted longer than 100 ms (Tinkhauser *et al.*, 2017*b*). Because of their higher frequency, gamma bursts are too short-lived to be captured in sufficient quantity by these criteria and were therefore not considered further for analyses. The OFF medication state may have also served to diminish and shorten gamma bursts, as LFP activity in this frequency band and related bursting depends on dopaminergic input (Brown *et al.*, 2001; Androulidakis *et al.*, 2007; Lofredi *et al.*, 2018). The temporal relationship between alpha and beta bursts was screened and overlapping bursts were removed (Fig. 3). Non-burst periods were defined as periods without alpha, beta, or low gamma bursts. To perform further analysis, a 100 ms duration time window was placed around the centre of every detected burst and non-burst period; feature extraction, selection, and limb movement classification was then performed on these 100-ms periods.

### Decoding algorithm

The Gaussian Naïve Bayes classifier MATLAB implementation was used to differentiate between the upper and lower limb movements (Friedman *et al.*, 1997; Rish, 2001; Hand and Yu, 2011). In this algorithm, features are conditionally independent random variables, given the class. The estimated posterior probability is equal to the prior probability multiplied by the likelihood. Naïve Bayes estimates the densities of the features within each class, and then it models posterior probabilities according to Bayes rule. That is, for all k=1,…,K,

(1) ^(Y=k|X1,.,XP) =πY=k . ∏j=1PP(Xj|Y=k)∑k=1KπY=k ∏j=1PP(Xj|Y=k)

Where Y is the random variable corresponding to the class index of a trial (e.g. which limb is being moved), $X_{1},.,X_{P}\;$ are the random features of a trial (e.g. the top six ranked features from The ReliefF feature selection algorithm), π(*Y = k*) is the prior probability that a class index is k , which was computed based on the relative frequency distribution of each class. Finally, it classifies a trial by estimating the posterior probability for each class, and then assigns the observation to the class yielding the maximum posterior probability.

### Statistical analyses

The main measure of interest was the area under the receiver operator characteristic curve (AUC) of the classifier based on the different spectral features of the LFP signal. This was taken as an indicator of task-related information. Statistical analyses were performed using MATLAB (2018, Mathworks, Natick, MA, USA) and SPSS (2017, IBM Corp., Armonk, NY, USA). The AUC values, features weight values, and average power of EMG signals were tested with MATLAB Lilliefors test to check whether they were normally distributed or not. Prior to inclusion in ANOVAs and subsequent *post hoc* tests, Box-Cox transformation was applied to transform non-normally distributed data. Greenhouse-Geisser correction was applied where Mauchly’s test of sphericity was significant. *Post hoc* tests were paired *t*-tests. All data are presented as means ± standard error of the mean (SEM), and all significance *P*-values in this article are corrected for multiple comparisons using the false discovery rate (FDR) method.

### Data availability

Data are available upon reasonable request to the corresponding author.

## Results

### Subthalamic activity contains information predictive of forthcoming movement

Patients were asked to make voluntary movements of one side following an imperative cue. Prior to the onset of each block patients were informed whether extension of the wrist or dorsiflexion of the ankle would be instructed by the go cue. Patients therefore knew the voluntary movement to be made even before the onset of the first imperative cue in the block. The order of the upper and lower limb blocks was counterbalanced. The cue was the appearance of a target on a PC screen and was the same irrespective of block type. Recordings were made simultaneously from the six segmented contacts of the directional electrode in the STN and these were analysed in non-overlapping 100-ms duration windows taken from different periods in the task. Spectral features were extracted for each window and the naïve Bayes classifier was used to classify serial 100-ms duration windows according to whether the wrist or ankle would eventually be moved.

**Figure 4:**
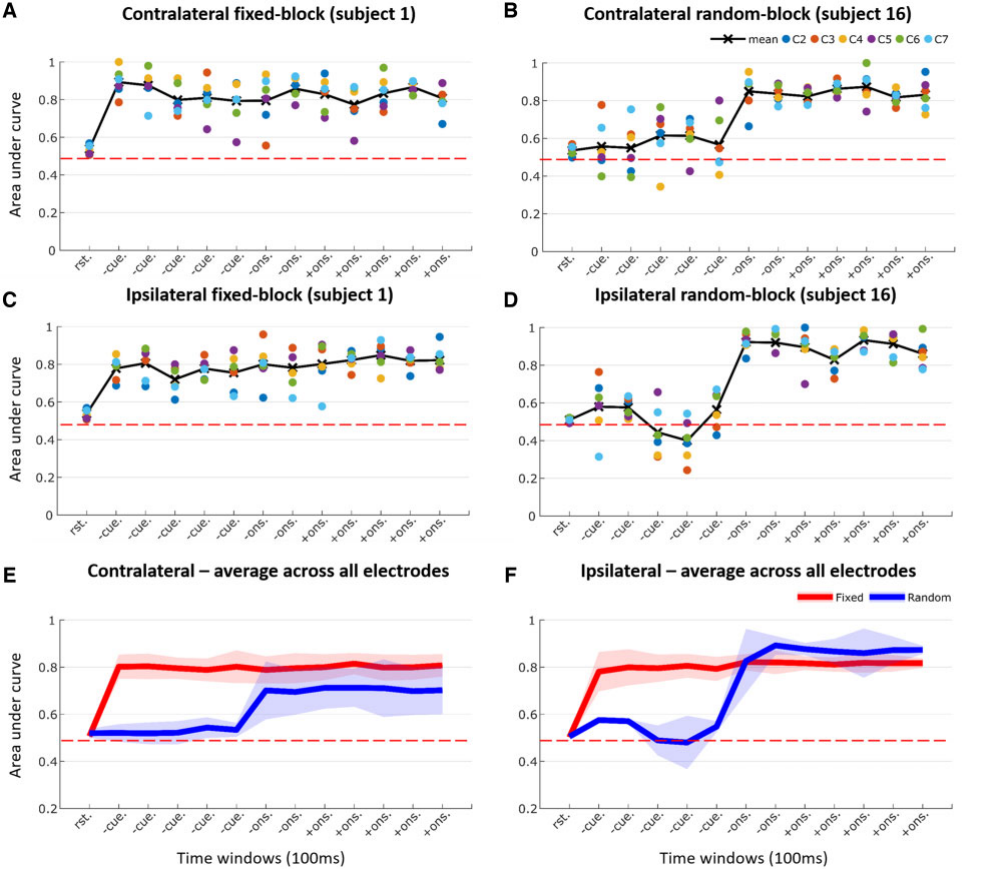
**Classification AUC across various task periods.** Task periods included: rest (rst.), pre-cue (-cue.), pre-movement onset (-ons.), and post-movement onset (+ons.). (**A**) Example of performance of one contralateral hemisphere during fixed-limb blocks. (**B**) Example of performance of one contralateral hemisphere during random-limb block. (**C**) Example of performance of one ipsilateral hemisphere during fixed-limb block. (**D**) Example of performance of one ipsilateral hemisphere during random-limb block. (**E**) Average across contralateral hemispheres during fixed-limb block (*n* = 23) and random-limb block (*n* = 6) across six directional contacts (c2–c7). (**F**) Average across ipsilateral hemispheres during fixed-limb (*n* = 13) and random-limb blocks (*n* = 2) across six directional contacts (c2–c7). **A** and **C** are for the left hemisphere of Subject 1 and **B** and **D** are for the left hemisphere of Subject 16. Horizontal dashed red line shows the AUC if classification were at chance level.

The STN contralateral to the movement was considered first. For each time point (100-ms window) AUC was averaged across the six directional electrode contacts. This is illustrated for an exemplar patient in Fig. 4A, and for group data across 23 electrodes in Fig. 4E. The mean AUC was 0.506 ± 0.013 at rest (prediction at chance levels). However, this increased to 0.798 ± 0.0396, 0.792 ± 0.0521 and 0.804 ± 0.0421 during the pre-cue, pre-movement onset and post-movement onset task periods, respectively (AUC averaged over windows and then across electrodes). An ANOVA of transformed AUC with main effect of period (rest, pre-cue, pre-movement onset and post-movement onset) was significant [*F*(1,22) = 206.544, *P* < 0.001]. *Post hoc* paired significance tests confirmed an increase in AUC in the pre-cue, pre-movement onset and post-movement onset periods compared to rest (*P* < 0.001, *P* < 0.001, *P* < 0.001, respectively). Therefore, the activity of neural ensembles picked up by the subthalamic electrode contained information about the nature of the forthcoming response to be actioned. Remarkably, prediction of the effector to be chosen began in the pre-cue period between instruction and imperative cue even though the transformed EMG average power during pre-cue [0.0058 ± 0.0135 arbitrary units (au)] was not different than that during rest (0.0035 ± 0.0074 au, *P* = 0.4006).

To confirm that the information available in the pre-cue period directly related to which limb was to be moved, we performed a control block in a group of four subjects (six electrodes) in whom there was no forewarning of the limb to be moved at the start of each block of trials. Here, the imperative cue instructed the limb to be moved, and this was randomized across trials in the same block. Under these circumstances the AUC during the pre-cue period was 0.5277 ± 0.0285, which was not different to the AUC during the preceding rest period (0.5198 ± 0.0194, *P* = 0.5143). Exemplar data from one subject are shown in Fig. 4B and data averaged across six electrodes are shown in Fig. 4E.

Next, we analysed ipsilateral voluntary movements. These were acquired in 8 of 18 subjects (13 electrodes; Fig. 4C and F), as recording was cut short in five patients because of fatigue. In subjects with paired contralateral and ipsilateral data the AUC was not significantly different between the two sides across any of the task periods. An ANOVA of transformed AUC with main effects task period (rest, pre-cue, pre-movement onset and post-movement onset) and side (contralateral and ipsilateral) confirmed a significant effect of period [*F*(1,12) = 178.7, *P* < 0.001], and a significant interaction between period and side [*F*(1.884,22.618)= 112.73, *P* < 0.001]. The effect of side was not significant [*F*(1,12) = 3.521, *P* = 0.085]. As before, the effect of task period was due to an increase in AUC on both ipsilateral and contralateral sides in the pre-cue, pre-movement onset and post-movement onset periods compared to rest (*P* < 0.001, *P* < 0.001, and *P* < 0.001, respectively). The interaction between period and side was not further investigated.

We also repeated our control random-limb blocks in two of the 18 subjects while they made ipsilateral movements (Fig. 4D and F). As before, there was no forewarning of the limb to be moved at the start of each block of trials. The imperative cue dictated the responding limb, and this was randomized across trials. Under these circumstances the AUC during the pre-cue period was 0.5325 ± 0.0278, which was not different to that during the preceding rest period (0.5054 ± 0.0058).

To summarize, STN activity over a wide band of frequencies carries significant information about intended contralateral and ipsilateral limb movements even when these are yet to be made, provided that prior instructions enable action selection.

### Bursts of 8–30 Hz subthalamic activity compromise the prediction of forthcoming movement

**Figure 5:**
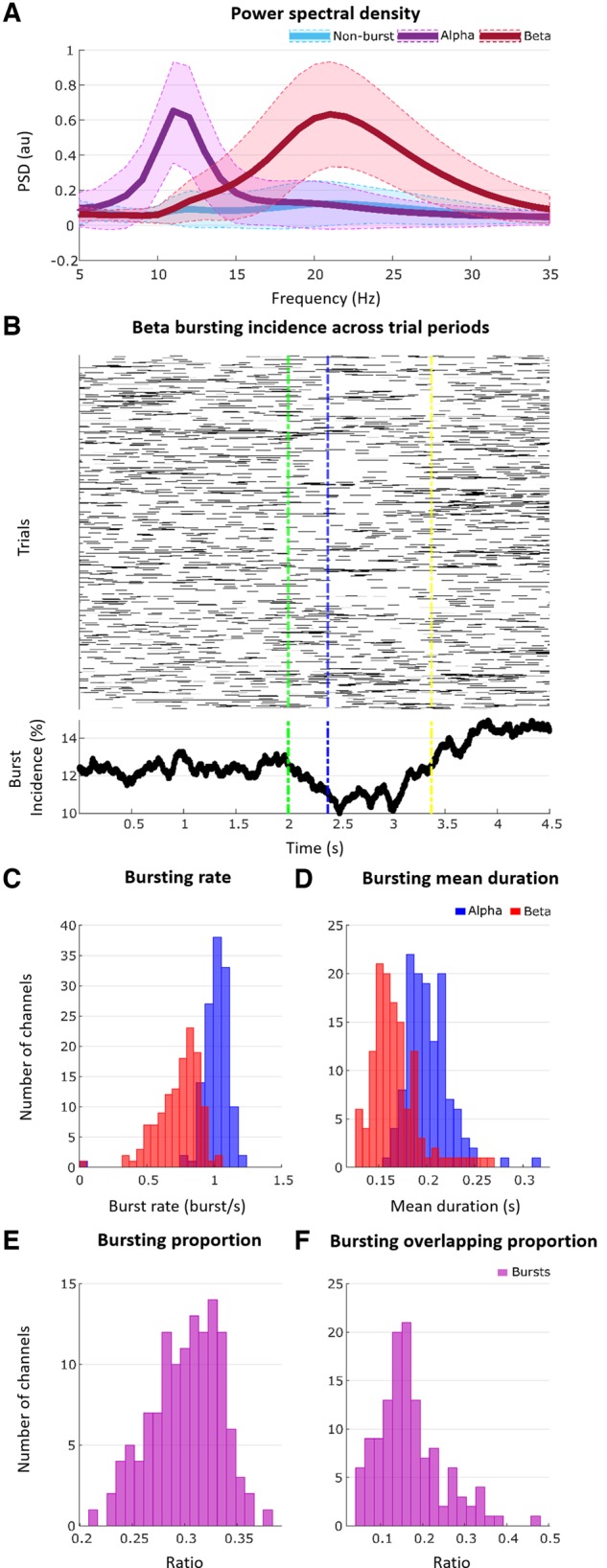
**Burst characteristics during the pre-cue period in the fixed-limb experiment.** (**A**) Example shows power spectral density for the three bursting states (non-burst, alpha, and beta) from the left hemisphere of Subject 1 (channel 4). (**B**) Raster plot of bursts (*top*) and mean of beta burst incidence (% of total trials; *bottom*) at each time point for all trials of fixed-limb blocks (23 hemispheres, 9801 trials in total) aligned to cue (green vertical line). Mean of trial movement onsets is blue vertical line and mean of trial movement offsets is yellow vertical line. (**C**) Histogram of burst rate for alpha (blue) and beta (red) bursts across all contralateral hemispheres (*n* = 23). The overlap between the two burst distributions is dark red. (**D**) Histogram of mean burst duration for alpha and beta bursts across all contralateral hemispheres (*n* = 23). (**E**) Histogram of the proportion of the whole pre-cue period taken up by the combined alpha and beta bursts across all contralateral hemispheres (*n* = 23). Ratio of 0.2 means, for example, that alpha and beta bursts comprise 20% of the pre-cue period. (**F**) Histogram of proportion of bursts that overlap between alpha and beta across all contralateral hemispheres (*n* = 23).

Our ultimate goal was to determine whether the information about future movement held by local circuits was compromised by bursts of locally synchronized activity, as expressed in the LFP (Fig. 5A). The incidence of bursts fell off steeply after the imperative cue (Fig. 5B), but bursts were still frequent during the pre-cue period during which the AUC for predictions of contralateral limb movement was already elevated in our standard forewarned paradigm (alpha burst rate: 1.0125 ± 0.1208 burst/s; alpha burst duration: 0.1672 ± 0.0259 s, beta burst rate: 0.7342 ± 0.159 burst/s, and beta burst duration: 0.2015 ± 0.0225 s; Fig. 5C and D).

Accordingly, 4 s of pre-cue period were reclassified with respect to whether they contained alpha (8–12 Hz) or beta (13–30 Hz) bursts or not. Alpha and beta bursts were detected first, then a 100-ms window was centred on the midpoint of each extracted burst. Periods representative of bursts in the beta frequency were selected that did not overlap with bursts in the alpha band, and vice versa (Fig. 3E). Histograms of proportion of the pre-cue period occupied by alpha and beta bursts (0.3007 ± 0.0328) and of the proportion of alpha and beta bursts that overlap (0.1699 ± 0.0772) for all the 23 contralateral hemispheres are shown in Fig. 5E and F, respectively. Naïve Bayes classifier was again used to classify alpha, beta and non-burst classified pre-cue sections according to whether the wrist or ankle would eventually be moved. AUCs were averaged across the three directional contacts on each electrode that afforded the best prediction. Averaging was performed separately for two contact configurations. In the first contact configuration, the AUC was averaged across the three burst states (non-burst, alpha burst and beta burst) for each of the six directional contacts and then the three contacts affording the best predictions as measured by the AUC defined (same three contacts, hereafter termed the three best contacts). In the second, the three best contacts were independently selected for each burst state (different three contacts). This was performed in case there was a shift in the spatial distribution in activities according to burst state, and indeed the mean AUC for the same three best contacts (0.6833 ± 0.0272) was less than that for the different three best contacts (0.7027 ± 0.0258, *P* < 0.001) in the non-burst state. Three, rather than six, directional contacts were considered, as when testing the effect of bursting we also wanted to explore the impact of bursting on feature weights, and it was pointless to include directional contacts where features contributed relatively little to classification.

**Figure 6:**
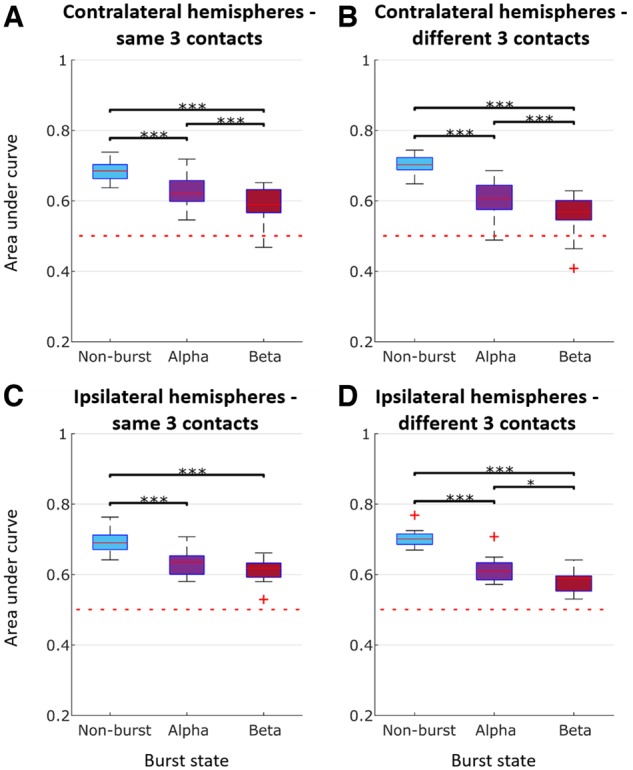
**Classification AUC during the pre-cue period for different burst conditions [alpha (8–12 Hz), beta (13–30 Hz), and non-burst].** (**A**) Average across all contralateral hemispheres (*n* = 23) for same best three contacts (i.e. the three contacts affording the best predictions as measured by the AUC). (**B**) Average across all contralateral hemispheres (*n* = 23) for different best three contacts. (**C**) Average across all ipsilateral hemispheres (*n* = 13) for same best three contacts. (**D**) Average across all ipsilateral hemispheres (*n* = 13) for different best three contacts. ^***^P < 0.001. Box and whisker plots with median (red horizontal line), 25th and 75th percentiles (bottom and top edges of the box, respectively), and outliers (red+) noted. Horizontal dashed red line shows the AUC if classification were at chance level.

The above revealed that the AUC for contralateral STNs was decreased for pre-cue 100-ms windows containing alpha and beta bursts compared with those that only contained non-burst periods, as illustrated for the group data in Fig. 6A and B, independently of whether the same or different three contacts were considered. An ANOVA of AUC for the same best three contacts during the pre-cue period with main effect of state confirmed the significance of this factor [*F*(2,42) = 65.1, *P* < 0.001]. *Post hoc* tests demonstrated that the AUC during non-burst windows exceeded that during alpha (*P* < 0.001) and beta bursts (*P* < 0.001), and the AUC in alpha bursts exceeded that in beta bursts (*P* < 0.001) in the pre-cue period of the standard forewarned task. A similar ANOVA for the different best three contacts configuration confirmed the significance of burst state [*F*(2,42) = 110.497, *P* < 0.001]. *Post hoc* tests demonstrated that the AUC during non-burst windows exceeded that during alpha (*P* < 0.001) and beta bursts (*P* < 0.001), and the AUC in alpha bursts exceeded that in beta bursts (*P* < 0.001). Note that as the difference in AUC between the STN LFP with and without alpha or beta bursts was assessed in the pre-cue period it was likely independent of any change in reaction time, which would arise after the period of evaluation.

We also repeated the above in the subgroup of 13 subjects in whom we tested both contralateral and ipsilateral voluntary movements. In these subjects with paired data the drop in AUC during alpha and beta bursts did not differ between the two sides (Fig. 6C and D). An ANOVA of AUC in the pre-cue period with main effects burst state (non-burst, alpha burst or beta burst) and side (contralateral and ipsilateral) for the same best three contacts confirmed a significant effect of state [*F*(2,24) = 59.978, *P* < 0.001], but showed no effect of side [*F*(1,12) = 0.054, *P* = 0.820], nor interaction between state and side [*F*(2,24) = 0.106, *P* = 0.899]. The AUC during non-burst periods exceeded that during alpha (*P* < 0.001) and beta bursts (*P* < 0.001). However, although the AUC in alpha bursts exceeded that in beta bursts this was no longer significant (*P* = 0.1277). An ANOVA of AUC with main effects state (non-burst, alpha burst or beta burst) and side (contralateral and ipsilateral) for the different best three contacts confirmed a significant effect of state [*F*(2,24) = 166.471, *P* < 0.001], and showed no effect of side [*F*(1,12) = 0.238, *P* = 0.634], nor interaction between state and side [*F*(2,24) = 0.546, *P* = 0.586]. As previously, the AUC during non-burst windows exceeded that during alpha (*P* < 0.001) and beta bursts (*P* < 0.001), and the AUC in alpha bursts exceeded that in beta bursts (*P* = 0.0212).

In summary, regardless of whether the STN contralateral or ipsilateral to movement, or the same or different best three contacts were considered, alpha and beta bursts compromised the AUC, with the effect of beta bursts being significantly greater.

### Spectral features over a wide frequency range contribute to limb prediction

**Figure 7:**
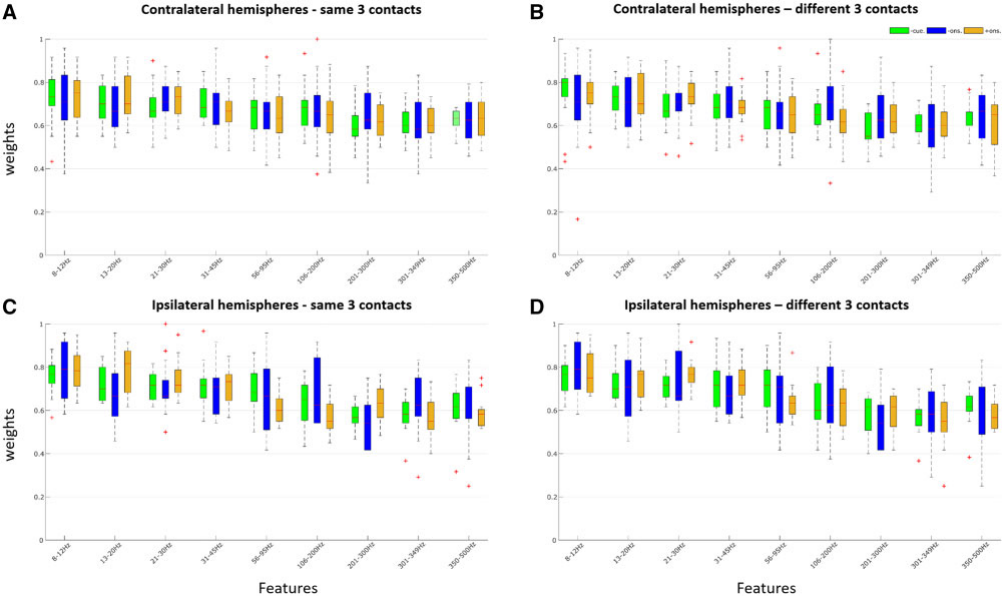
**Feature importance for prediction during different task periods in fixed-limb blocks for raw LFP before classification into burst conditions.** (**A**) Average across all contralateral hemispheres (*n* = 23) for the same best three contacts in different task periods. Feature importance was correlated across task periods e.g. between pre-cue and pre-movement onset, Spearman’s rho = 0.833, *P* = 0.008, between pre-cue and post-movement onset, Spearman’s rho = 0.933, *P* < 0.001, and between pre- and post-movement onset, Spearman’s rho = 0.85, *P* = 0.006. (**B**) Average across all contralateral hemispheres (*n* = 23) for the different best three contacts. Feature importance was correlated across task periods e.g. between pre-cue and pre-movement onset, Spearman’s rho = 0.87, *P* = 0.003, between pre-cue and post-movement onset, Spearman’s rho = 0.862, *P* = 0.004, and between pre- and post-movement onset, Spearman’s rho = 0.917, *P* = 0.0013. (**C**) Average across all ipsilateral hemispheres (*n* = 13) for the same best three contacts. Feature importance was correlated across task periods e.g. between pre-cue and pre-movement onset, Spearman’s rho = 0.783, *P* = 0.0172, between pre-cue and post-movement onset, Spearman’s rho = 0.767, *P* = 0.021, and between pre- and post-movement onset, Spearman’s rho = 0.467, *P* = 0.2125. (**D**) Average across all ipsilateral hemispheres (*n* = 13) for the different best three contacts. Feature importance was correlated across task periods e.g. between pre-cue and pre-movement onset, Spearman’s rho = 0.967, *P* < 0.001, between pre-cue and post-movement onset, Spearman’s rho = 0.967, *P* < 0.001, and between pre- and post-movement onset, Spearman’s rho= 0.95, *P* < 0.001. Task periods were pre-cue (-cue.), pre-movement onset (-ons.), and post-movement onset (+ons.).

Spectral features drawn across a wide frequency band (8–500 Hz) contributed to prediction of the limb to be moved. This was true for predictions made with the LFP signal during the pre-cue, pre-movement onset and post-movement onset periods of the fixed-limb paradigm before classification into burst states, as indexed by feature weights (Fig. 7). Moreover, feature weights were significantly and strongly correlated across task periods (Fig. 7 legend).

**Figure 8:**
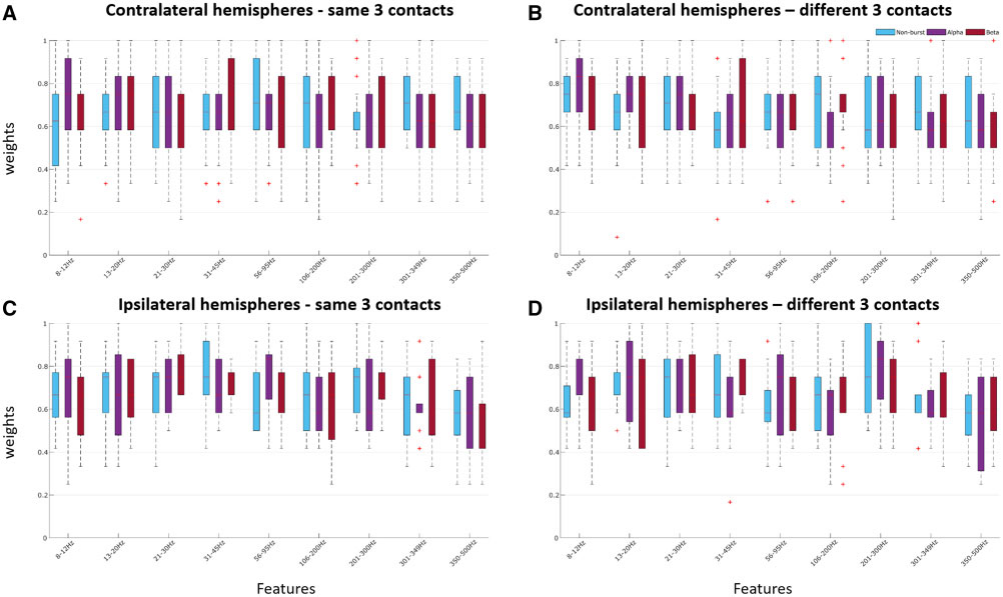
**Feature importance during the pre-cue period for the burst conditions: non-bursts, alpha bursts, and beta bursts.** (**A**) Average across all contralateral hemispheres (*n* = 23) for the same best three contacts. (**B**) Average across all contralateral hemispheres (*n* = 23) for the different best three contacts. (**C**) Average across all ipsilateral hemispheres (*n* = 13) for the same best three contacts. (**D**) Average across all ipsilateral hemispheres (*n* = 13) for the different best three contacts.

The same was also true of predictions made with the LFP signal during the pre-cue period of the fixed-limb paradigm after separation into burst states (non-burst, alpha, and beta bursts) (Fig. 8). Overall, the importance of features was similar between task periods (Fig. 7), but less between burst states (Fig. 8).

Finally, in an exploratory analysis we investigated whether bursts weakened feature weights. We focused on those features that were consistent with high frequency oscillations suspected of being related to local multi-unit activity (features > 300 Hz; Meidahl *et al.*, 2019). An ANOVA of transformed high frequency (>300 Hz) feature weights for the same best three contralateral contacts configuration during the pre-cue period with main effect of burst state showed a significant effect of burst state [*F*(2,42) = 17.87, *P* < 0.001]. *Post hoc* tests demonstrated that the high frequency weights during non-burst windows exceeded those in alpha bursts (*P* < 0.001) and beta bursts (*P* < 0.001). Another ANOVA of transformed high frequency feature weights for the different best three contralateral contacts configuration also demonstrated that burst state was a significant main effect [*F*(2,42) = 5.482, *P* = 0.008]. *Post hoc* tests demonstrated that the high frequency weights during non-burst windows exceeded those during alpha (*P* = 0.0193) and beta bursts (*P* = 0.0159).

## Discussion

Our findings demonstrate that low frequency (8–30 Hz) bursts of activity in the STN LFP impair the LFP-based prediction of future limb selection during voluntary movements. This is consistent with the hypothesis that the increased synchronization denoted by bursting compromises information coding capacity in basal ganglia networks (Brittain and Brown, 2014). This effect was maximal in the beta frequency (13–30 Hz) band. Bursts in this band are attenuated and shortened in duration by treatment with levodopa in patients with Parkinson’s disease (Tinkhauser *et al.*, 2017*b*), and specifically shutting down long duration bursts using beta-triggered adaptive DBS also leads to improvement in motor impairment (Tinkhauser *et al.*, 2017*a*). Moreover, the incidence of such bursts correlates with clinical scores of bradykinesia-rigidity (Tinkhauser *et al.*, 2017*a*, *b*) and with the velocity of specific movements (Torrecillos *et al.*, 2018; Lofredi *et al.*, 2019). Together, these observations suggest that bursts of beta activity may be a key abnormality in the parkinsonian state (Deffains and Bergman, 2019), and the current findings provide a candidate mechanism for their effect and indicate that beta bursts restrict information coding capacity in basal ganglia networks.

The STN bursts are themselves likely to reflect synchronized oscillatory input, given that LFP activities of lower frequency are thought to reflect afferent activity (Buzsáki *et al.*, 2012). In the case of the STN, although LFP beta activity is locally generated (Kühn *et al.*, 2005; Weinberger *et al.*, 2006; Trottenberg *et al.*, 2007; Marmor *et al.*, 2017), it is coherent with, and led by, cortical beta oscillations consistent with cortical driving (Fogelson *et al.*, 2005; Hirschmann *et al.*, 2011; Litvak *et al.*, 2010; Cagnan *et al.*, 2019). However, bursts compromised the feature weights of activities above 300 Hz, which are believed to be generated within the STN and which have recently been linked to multi-unit activity and phase-amplitude coupling (Foffani *et al.*, 2003; López-Azcárate *et al.*, 2010; van Wijk *et al.*, 2016, 2017; Meidahl *et al.*, 2019). Indeed, as the latter is an interaction with the phase of local beta activity (López-Azcárate *et al.*, 2010), and is focused in beta bursts (Meidahl *et al.*, 2019), it may be that phase-amplitude coupling is a manifestation of the limitation in information coding capacity exerted by relatively sustained periods of increased, afferent driven, beta synchronization. Microelectrode studies might help confirm whether incoming low frequency bursts serve to limit information coding within the STN, which then impacts on STN output in the form of multi-unit activity.

Although bursting denotes increased synchronization (Levy *et al.*, 2002*a*, *b*; Kühn *et al.*, 2005; Weinberger *et al.*, 2006; Mallet *et al.*, 2008; Deffains *et al.*, 2018) and excessive synchronization may potentially limit information coding capacity in basal ganglia networks (Mallet *et al.*, 2008; Brittain and Brown, 2014), it should be acknowledged that low frequency bursting might exert its effects through some alternative unforeseen mechanism. Regardless, however, the effects shown here and the presence of physiological beta bursts in cortical-basal ganglia circuits in the healthy state raise the possibility that the information regarding future voluntary movement in motor circuits is dynamic and punctuated by low frequency bursts that serve to temporarily degrade information favouring one or other course of action. This process, which might under normal circumstances allow for flexibility in response, continues until the imperative cue, when bursting is relatively suppressed and the motor system fully commits to a given action. Of note, time-limited relative increases in beta activity in the STN have previously been linked with the need to delay while more evidence is accumulated when making difficult decisions about action choices (Herz *et al.*, 2018).

It is also worth considering what process might underlie the change in neural activity allowing prediction of forthcoming movement in the first place. In the fixed-limb blocks reliable predictions could be derived from the STN LFP even before presentation of the imperative cue, provided that the subject was forewarned about the limb to be moved. This predictive activity could reflect anticipatory action selection, anticipatory invigoration of the motor response or subtle postural changes made to facilitate the behavioural response when it would eventually be needed. However, the absence of a significant difference in background EMG activity between the rest and pre-cue periods would be against the STN changes denoting an instruction-driven shift in posture or subliminal movement. The observation that LFPs from both the contralateral and ipsilateral STN were equally predictive of the limb to be moved is interesting and may relate to the fact that patients were recorded OFF medication. Lateralized movement-related reactivity effects in the LFP are dependent on dopaminergic therapy (Androulidakis *et al.*, 2007). However, the finding of predictive LFP activity in both STN does not necessarily mean that the activities within the two STN are identical.

The effect of beta bursts upon information coding capacity was demonstrated for the pre-cue period. However, the incidence of beta bursts dropped after presentation of the go cue and during the movement, so how might a burst related limitation of information coding explain motor impairment? We have previously shown that, although beta bursts appear less frequently after cues instructing movement, they do still occur in some responses and it is these responses that have slower peak velocities in patients with Parkinson’s disease (Torrecillos *et al.*, 2018; Tinkhauser *et al.*, 2020, in press). Similarly, beta bursts can reoccur during self-paced repetitive upper limb movements and during gait, and do so in association with bradykinesia and gait freezing, respectively (Anidi *et al.*, 2018; Lofredi *et al.*, 2019). Studies of beta-triggered adaptive DBS also provide evidence that beta bursts occur during movement. Two such studies have assessed repeated reach and return movements (Johnson *et al.*, 2016; Iturrate *et al.*, 2019). They indicate that 30–70% of trials involve the delivery of adaptive DBS at any given point in the movement trajectory. As stimulation is triggered by beta bursts this serves as a surrogate measure for the percentage of trials with beta bursts at any given point in the movement trajectory. Studies of beta-triggered adaptive DBS also provide some support for a causal link between beta bursts and motor impairment, in so far as stimulation cuts short beta bursts while improving kinematic measures of repeated tapping and wrist movements (Tinkhauser *et al.*, *2017a*, *b*; Piña-Fuentes *et al.*, 2019; Velisar *et al.*, 2019).

Alpha band bursts also significantly impaired prediction, albeit not as strongly as beta band bursts. This is interesting as it suggests that increased synchronization need not necessarily be limited to the beta band to have a deleterious effect. It also has its corollary in clinical studies that often consider the correlations between motor impairment and STN LFP activities in a combined alpha-beta band (Kühn *et al.*, 2006, 2009; Chen *et al.*, 2010; Neumann *et al.*, 2016). A further study has demonstrated a positive correlation between STN sub-beta frequency oscillations and axial symptoms (Sharott *et al.*, 2014). Alternatively, the alpha band effects seen in the present study might just represent spectral leakage from a dominant effect in the beta band, particularly as some evidence points to the lower frequencies in the beta band as being most linked to motor impairment. For example, it is these that are exaggerated in amplitude in Parkinson’s disease relative to obsessive compulsive disorder patients also recorded in the dorsolateral STN, and which are suppressed by levodopa in the same patients. In contrast, LFP activities at higher frequencies in the beta band are more similar in amplitude in this region between the two conditions (Rappel *et al.*, 2018).

The current findings are also of interest in that they suggest that local STN activity may potentially be decoded to enable effector selection, in addition to force control (Mamun *et al.*, 2015; Tan *et al.*, 2016; Golshan *et al.*, 2018) in brain-machine interface applications. Information about the effector to be selected was present well in advance of the voluntary movement, affording hope that a similar control signal might be present in the STN in the absence of physical movement in paralysed patients or those with amputation. As already noted, LFPs from both the contralateral and ipsilateral STN were equally predictive of the limb to be moved, although predictions of force are better achieved from the contralateral STN (Tan *et al.*, 2016). Here, it is probably relevant that we recorded LFP activity with directional electrodes that allow a higher resolution sampling of local activity than conventional quadrapolar DBS electrodes (Zhang *et al.*, 2018). Under these circumstances we were able to predict the selected limb with an AUC of ∼0.8 from single time windows of 100-ms duration when averaging across three electrode contacts. It is likely that predictions could be strengthened still further if information were derived from several windows and the spatial patterning of activity across the six different directional contacts considered.

We should acknowledge several limitations of the current experiments. These were performed intraoperatively and hence the number of movement trials and blocks were limited. Features were therefore necessarily selected on empirical grounds and could not be independently identified through advanced machine learning algorithms. Predictions might have been improved with such algorithms, and also had recordings been achieved in chronically implanted patients, in whom any acute stun effects (Chen *et al.*, 2006) compromising STN processing would have abated.

In conclusion, using an STN LFP-based machine learning approach we have been able to distinguish forthcoming upper limb from lower limb movements and have shown that the predictive information within the LFP is compromised during bursts of alpha and beta activity, with the major effect occurring in the latter frequency band. These results support the hypothesis that bursts of low frequency activity in the STN restrict the overall capacity of the system to encode physiologically relevant information about intended actions. Given that such bursts are exaggerated in Parkinson’s disease, our findings provide mechanistic insight into the pathological relevance of beta dynamics, while also encouraging consideration of the STN as an alternative or complementary signal source in restorative brain-computer interfaces.

## Supplementary Material

awz417_Supplementary_Table

## Abbreviations

AUC =: area under the receiver operator characteristic curve
DBS =: deep brain stimulation
LFP =: local field potential
STN =: subthalamic nucleus
