# Supplementary material for: Subthalamic nucleus activity dynamics and limb movement prediction in Parkinson’s disease
Source: Brain. 2020 Feb 10;143(2):582–96. doi: 10.1093/brain/awz417 (PMC7009471; doi:10.1093/brain/awz417)
Supplement: awz417_Supplementary_Table [file awz417_supplementary_table.docx]

# Table 1 Clinical details. Num = number; Hem = hemisphere; R = right; L = left; y = years; m = male; f= female; MDS-UPDRS = Movement Disorder Society - Unified Parkinson’s disease rating scale Part III; SEM = standard error of the mean.

| **Subject** | **Sex** | **Age DBS (y)** | **Disease Duration (y)** | **Dominant Symptom** | **MDS-UPDRS OFF** | **MDS-UPDRS ON** | **Num Hem** | **STN site** | **Limb assessment** |
| --- | --- | --- | --- | --- | --- | --- | --- | --- | --- |
| 1 | m | 49 | 8 | akinetic rigid, left | 36 | 14 | 1 | L | ContraLat, IpsiLat |
|  |  |  |  |  |  |  | 2 | R | ContraLat, IpsiLat |
| 2 | m | 55 | 3 | akinetic-rigid right | 40 | 25 | 3 | L | ContraLat, IpsiLat |
|  |  |  |  |  |  |  | 4 | R | ContraLat, IpsiLat |
| 3 | f | 73 | 11 | akinetic-rigid right | 24 | 14 | 5 | L | ContraLat, IpsiLat |
|  |  |  |  |  |  |  | 6 | R | ContraLat, IpsiLat |
| 4 | m | 64 | 16 | akinetic-rigid, left | 56 | 23 | 7 | L | ContraLat |
|  |  |  |  |  |  |  | 8 | R | ContraLat, IpsiLat |
| 5 | m | 53 | 6 | akinetic-rigid, left | 30 | 13 | 9 | L | ContraLat |
| 6 | m | 34 | 7 | akinetic-rigid, right | 29 | 10 | 10 | L | ContraLat, IpsiLat |
| 7 | m | 54 | 11 | akinetic-rigid, left | 61 | 27 | 11 | L | ContraLat, IpsiLat |
|  |  |  |  |  |  |  | 12 | R | ContraLat, IpsiLat |
| 8 | m | 61 | 9 | akinetic-rigid, right | 30 | 6 | 13 | L | ContraLat |
|  |  |  |  |  |  |  | 14 | R | ContraLat |
| 9 | m | 72 | 9 | tremor dominant, right | 50 | 14 | 15 | L | ContraLat |
|  |  |  |  |  |  |  | 16 | R | ContraLat |
| 10 | f | 53 | 5 | akinetic rigid, right | 27 | 21 | 17 | R | ContraLat, IpsiLat |
| 11 | m | 64 | 13 | akinetic rigid, left | 40 | 7 | 18 | L | ContraLat, IpsiLat |
|  |  |  |  |  |  |  | 19 | R | ContraLat |
| 12 | f | 70 | 9 | tremor dominant, right | 30 | 6 | 20 | R | ContraLat, IpsiLat |
| 13 | f | 60 | 8 | akinetic-rigid, right | 41 | 16 | 21 | L | ContraLat |
|  |  |  |  |  |  |  | 22 | R | ContraLat |
| 14 | f | 69 | 16 | akinetic-rigid, right | 25 | 3 | 23 | L | ContraLat |
|  |  |  |  |  |  |  | 24 | R | ContraLat |
| 15 | m | 65 | 10 | akinetic-rigid, left | 38 | 24 | 25 | R | ContraLat, IpsiLat |
| 16 | m | 61 | 8 | akinetic-rigid,  left | 34 | 15 | 26 | L | ContraLat, IpsiLat |
| 17 | m | 67 | 21 | akinetic-rigid, left | 30 | 10 | 27 | L | ContraLat |
|  |  |  |  |  |  |  | 28 | R | ContraLat |
| 18 | m | 43 | 11 | akinetic-rigid, right | 31 | 11 | 29 | L | ContraLat |
| **Mean ± SEM** | **m(13), f(5)** | **59.3 ± 10.4** | **10.1 ± 4.3** |  | **36.2 ± 10.4** | **14.4 ± 7.1** |  |  |  |
